# Observation or Otolaryngology Surveillance After Ventilation Tube Insertion in Children: The ConVenTu Noninferiority Randomized Clinical Trial

**DOI:** 10.1001/jamaoto.2025.2880

**Published:** 2025-10-09

**Authors:** Rikki Yahiro, Bjarne Austad, Anne-S. Helvik, Ann Helen Nilsen, Øyvind Salvesen, Wenche Moe Thorstensen

**Affiliations:** 1 Department of Neuromedicine and Movement Science, The Norwegian University of Science and Technology (NTNU), Trondheim, Norway; 2 Department of Otolaryngology, Head and Neck Surgery, St Olav’s University Hospital, Trondheim, Norway; 3 General Practice Research Unit, Department of Public Health and Nursing, Norwegian University of Science and Technology (NTNU), Trondheim, Norway; 4 Øya Medical Centre, Trondheim, Norway; 5 Department of Public Health and Nursing, Norwegian University of Science and Technology (NTNU), Trondheim, Norway

## Abstract

**Importance:**

Middle-ear ventilation tube (VT) insertion is the most common ambulatory surgery in children. Follow-up for these patients may continue for 2 or more years and consume a great deal of health care resources. Research into the appropriate level of care for such controls is lacking.

**Objective:**

To determine whether observation by general practitioners (GPs) yielded noninferior audiometric outcomes 2 years after VT insertion compared to otorhinolaryngology (ear, nose, and throat [ENT]) follow-up by specialist health care services.

**Design, Setting, and Participants:**

In the multicenter ConVenTu (Control of Ventilation Tubes) noninferiority randomized clinical trial, patients from 6 Norwegian ENT departments were assessed for eligibility from August 15, 2017, to August 30, 2021. Children between 3 and 10 years of age requiring VT insertion were eligible for the study. Patients with comorbidities or hearing loss exceeding 50 dB in at least 1 ear were excluded. The data were analyzed between February 19, 2024, and May 24, 2024.

**Intervention:**

Observation by the patient’s GP.

**Main Outcomes and Measures:**

The primary outcome was the difference between the means of pure-tone averages (PTAs) measured at inclusion and 2 years after VT insertion. Children were randomized in blocks of varying sizes with an allocation ratio of 1:1 stratified by study center. Differences were compared between observation and ENT follow-up groups using linear mixed model analysis. Analyses were by intention-to-treat, and a margin of 5 dB was used in the determination of noninferiority. The audiographers responsible for audiometric testing were blinded to patient allocation. Secondary outcomes included audiometric and tympanometric resolution as well as complications registered at control.

**Results:**

A total of 305 children requiring VT insertion (median [IQR] age, 4 [3-6] years; 185 [60.7%] male) were included in the trial (153 in GP arm and 152 in ENT arm). Of these, 145 children in each group completed audiometric control at 2 years. Audiometric outcomes of the observation group were found to be noninferior to those who routinely followed up with ENT (0.16 dB [1-sided 97.5% CI lower bound, −1.52 dB]) 2 years after VT insertion, well within the noninferiority threshold of 5 dB. There was also no difference in the total number of patients with complications (odds ratio, 0.67 [1-sided 97.5% CI lower bound, 0.39]).

**Conclusion and Relevance:**

In this randomized clinical trial, postoperative observation in general practice was noninferior to routine follow-up of patients who underwent VT surgery in Norway in terms of audiometric outcomes 2 years after VT insertion in otherwise healthy children. These findings may help guide the allocation of resources in the follow-up of these patients.

**Trial Registration:**

ClinicalTrials.gov Identifier: NCT02831985

## Key Points

**Question:**

Is observation by general practitioners a sufficient alternative to routine otorhinolaryngology (ear, nose, and throat [ENT]) follow-up for the postoperative care of children with middle-ear ventilation tubes (VTs) in terms of audiometric outcomes?

**Findings:**

In the multicenter ConVenTu (Control of Ventilation Tubes) noninferiority randomized clinical trial including 290 children between 3 and 10 years of age, hearing outcomes 2 years after VT insertion were noninferior in children assigned to observation by their general practitioner compared to those who underwent routine follow-up through an ENT department.

**Meaning:**

Observation without routine control yielded noninferior audiometric outcomes when compared to routine ENT follow-up 2 years after VT insertion.

## Teaser

This noninferiority randomized clinical trial compared audiometric outcomes 2 years after the insertion of middle-ear ventilation tubes in children who were observed by general practitioners vs those routinely followed up by otorhinolaryngology specialists.

## Introduction

The insertion of middle-ear ventilation tubes (VTs) is one of the most performed ambulatory surgeries in children.^1^ This is done by making a small incision in the tympanic membrane and inserting a small tube or grommet to allow for ventilation of the middle ear.^2^ The most common indication for VT treatment is otitis media with effusion (OME).^2^ OME is defined as middle-ear effusion without signs of infection^3^ and is the most common cause of impaired hearing in children.^4^ Other indications include recurring acute otitis media and infections that persist despite antibiotic therapy.^2^ In an epidemiological study performed in 2020, 25% to 30% of children with frequent ear infections had been treated with VTs.^5^ In Norway, 6700 of these procedures are performed annually (128 of 100 000 individuals).^6^ In 2010, in the US, an estimated 413 000 VT insertions were performed.^7^

Postoperative follow-up of these patients has traditionally been performed by the operating physician or through the otorhinolaryngology (ear, nose, and throat [ENT]) department responsible for VT insertion.^2^ Controls are meant to measure postoperative outcomes and to allow for the early detection of complications should they arise.^2,8^ Complications are generally transient or cosmetic in nature, such as otorrhea, tube extrusion or obstruction, tympanosclerosis, or retraction.^9^ Serious complications such as medial displacement of VTs into the middle ear, or cholesteatoma, are exceedingly rare.^9^

The latest American Academy of Otolaryngology–Head and Neck Surgery Foundation (AAO-HNS) guidelines recommend follow-up by the surgeon/designee 3 months after VT insertion.^2^ With subsequent follow-up in 6-month intervals, regardless of how well the child is doing, over 2 to 3 years or until spontaneous VT extrusion.^2^ In contrast to this, the National Institute for Health and Care Excellence (NICE) guidelines recommend audiometric control 6 weeks after surgery and further follow-up for 1 year if hearing loss has resolved.^10^ Despite these guidelines, only approximately one-quarter of patients attend follow-up until tube extrusion,^1^ which usually occurs spontaneously within 18 months of insertion.^2,9^ Both the NICE and AAO-HNS guidelines stress the importance of further research surrounding the follow-up of these patients.^2,11^

General practitioners (GPs) have previously been suggested as surrogates for postoperative follow-up due to the ease of access and continuity of care they provide.^12^ In Norway, contact with the specialist health care system is made by either referral or prior appointment, with GPs acting as gatekeepers.^13^ As such, GPs are the first line of contact for patients in need of care. A retrospective study found no differences in hearing outcomes between patients undergoing VT surgery who received follow-up by GPs compared to specialist health care services.^14^ However, the low sample size and lack of randomization made it difficult to draw conclusions.^14^ The ConVenTu (Control of Ventilation Tubes) trial was therefore designed and executed to compare hearing outcomes 2 years after VT insertion of patients who underwent routine follow-up by specialist health care services when compared to those with no routine follow-up and contact with their GP as required.^15^

## Methods

### Trial Design and Participants

The multicenter ConVenTu trial was a noninferiority randomized clinical trial with data collection between August 2017 and November 2023. Children were assessed for eligibility from 6 hospitals in Norway from August 15, 2017, to August 30, 2021, and participants were followed up by either the clinic where they underwent the surgery or with their GP. The Department of Otolaryngology, Head and Neck Surgery, at St Olav’s University Hospital was the primary investigating center and was responsible for coordinating independent personnel at each trial site. Ethical approval was obtained through the Regional Committee for Medical and Health Research Ethics in Mid-Norway. The trial protocol is both published^15^ and available in Supplement 1. The parents or guardians of the included children provided informed written consent. Children refusing participation were not included. The Consolidated Standards of Reporting Trials (CONSORT) reporting guideline was followed.

Patients were recruited by participating clinicians through the outpatient clinics of the participating centers. Eligible candidates were children between 3 and 10 years of age requiring VT treatment. The final decision of indication for VT insertion was made by the operating physician. Patients who received prior VT treatment were eligible for inclusion in this study. Exclusion criteria included medical syndromes or severe comorbidities, a lack of comprehension of the Norwegian language, or a hearing loss of greater than 50 dB in at least 1 ear. Demographic data, including sex, were collected using questionnaires filled out by the parents or guardians of the included children.

### Randomization

Patients were randomized to either routine ENT follow-up or observation by their GP on the day of VT insertion. Randomization was performed in blocks of varying sizes with an allocation ratio of 1:1 stratified by study center. The process was carried out by a nurse using the WebCRF software developed by the Unit for Applied Clinical Research at the Faculty of Medicine and Health Sciences, Norwegian University of Science and Technology (NTNU).^16^

Blinding of patients, treating physicians, and parents was not possible due to the nature of the trial. The certified audiologists responsible for audiometric testing at inclusion and 2 years after insertion were blinded to patient allocation. Participants were assigned a number indicating group, which was unknown to both the trial statistician and outcomes assessor until data analyses were completed.

### Procedures

Eligible patients were marked for inclusion by participating clinicians and informed of the trial after their initial outpatient assessment. Each child was subject to audiometry, tympanometry, and clinical assessment by an otolaryngologist or ENT resident prior to inclusion. The use of pure tone, play, or visual reinforcement audiometry was dependent on the age and patient cooperation. No additional guidelines, outside of those internationally available, related to indication for operative intervention or choice of VT type were used for this study. Operative indication was decided by and reported by the operating surgeon alongside previous surgical history and type of VT inserted.

Postoperative discharge documents were sent electronically to the patients’ GPs. Discharge documents sent to the GPs of patients not allocated to routine follow-up included a guideline for how to treat the most common complications (eAppendix in Supplement 2). No special training was provided to GPs outside of these guidelines. GPs were informed that their referrals to ENT would be prioritized should they require assistance. Parents were asked to contact their child’s GP should complications arise and to book an appointment 18 months after VT insertion. This 18-month follow-up appointment was intended to assess whether VTs were still present, allowing for referral to ENT for removal in keeping with current practice.

Patients allocated to ENT follow-up received an appointment 6, 12, and 18 months after surgery. Both routine follow-up and observation groups received an appointment at their respective ENT departments 2 years after VT insertion. Here, they underwent tympanometry and audiometry in addition to clinical examination, including otomicroscopy. The examining ENT physician recorded complications and audiometry results and referred the patient to surgery for the surgical extraction of VTs if indicated. Complications were recorded by filling in a form where retraction, perforation, recurrent OME, and cholesteatoma were options, as well as whether VTs were present at the 2-year control. This form was divided to allow for reporting for both ears. An additional free-text section allowed for further specification or reporting of other complications. Only data regarding referral to extraction were collected, and not data related to the surgical procedure itself.

### Main Outcomes

The primary outcome was hearing measured 2 years after VT insertion compared to baseline. The difference in hearing from baseline to 2-year control between patients undergoing routine follow-up through an ENT department was compared to those in the observation group to assess noninferiority. Hearing was assessed using the pure-tone average (PTA), which is calculated by averaging hearing thresholds measured at 0.5 Hz, 1 kHz, 2 kHz, and 4 kHz through pure-tone audiometry, ear-specific play audiometry, or ear-specific visual reinforcement audiometry. Missing frequencies were not imputed. The patients who were missing frequencies were included in the analysis, with averages calculated for the number of available frequencies. The measurement of hearing thresholds is performed in 5 dB intervals,^17^ and we considered 5 dB the minimum change of clinical significance.^15^ Therefore, an absolute difference in mean PTA between groups of less than 5 dB was considered equivalent.

Secondary outcomes measured included number and type of complications present at 2-year control, the number of reoperations, and audiometric and tympanometric resolution. Audiometric resolution was defined as patients with PTA values less than or equal to 20 dB at 2-year control. Tympanometric resolution was defined as the proportion of patients with an amelioration of tympanometric outcomes (went from a B curve to a C curve or A curve^18^) at 2-year control when compared to at inclusion.

### Statistical Analysis

The absolute minimum number of participants required for 95% power at a 2-sided significance level of 5% and a difference in means of at least 5 dB was determined to be 105 per group in a power analysis performed prior to trial start. An additional 30 participants per trial arm were added to adjust for the 6 centers, applying a general rule of thumb of at least 10 patients per parameter. Additional participants were originally intended to be included to account for multiple factors, such as skew distribution and dropouts, with an assumed dropout rate of 20%. The trial planned to include 200 patients per group. However, due to a dramatic drop in VT insertion during the COVID-19 pandemic, truncation and adjustment for dropouts were abandoned. Measures were taken surrounding the 2-year control, where study staff would contact patients and remind them of their follow-up appointments to keep the dropout rate as low as possible. The resulting revision yielded a minimum requirement of 135 patients in each arm. A total of 105 patients were needed to yield 95% power with a minimum difference in means of 5 dB using a 1-sided significance level of 2.5% due to the testing of noninferiority and an additional 5 patients per study center per arm of the trial.

All analyses were performed by intention-to-treat. In the case of planned unilateral VT insertion, the healthy ear was excluded from analysis. Data were not imputed for those lost to follow-up. Outcomes compared between the ENT follow-up and observation groups after 2 years were compared using a linear mixed model (LMM), with the combination of time and treatment as fixed factors, and participant identifier and unilateral VT insertion as random factors. Results of audiometric outcomes are presented with a 1-sided 97.5% CI.

The number of complications was compared by frequency of occurrence between groups. Odds ratios (ORs) were used to determine effect size and are presented with their corresponding 1-sided 97.5% CIs. Standing VTs at control were not considered a perforation for this analysis. Audiometric resolution was determined by assessing the proportion of patients who ended the trial with a PTA of 20 dB or less and comparing them across groups. Tympanometric resolution compared the proportion of those with the amelioration of 1 or more B curves at the time of control across groups. Patients missing tympanometric data at inclusion or conclusion, those with persistent VT or perforation, and those lacking B curves both at inclusion and conclusion were excluded from this analysis.

Participants were divided into subgroups based on age at inclusion, hearing measured in PTA at inclusion, and sex, and results were compared across groups by *t* test.

A detailed statistical analysis plan was produced and signed prior to the completion and locking of the trial database (Supplement 1). No interim analyses were performed. Data analyses were performed using R, version 4.3.1 (R Project for Statistical Computing) and STATA, version 18 MP (StataCorp LLC), between February 19, 2024, and May 24, 2024.

## Results

**Figure 1.**
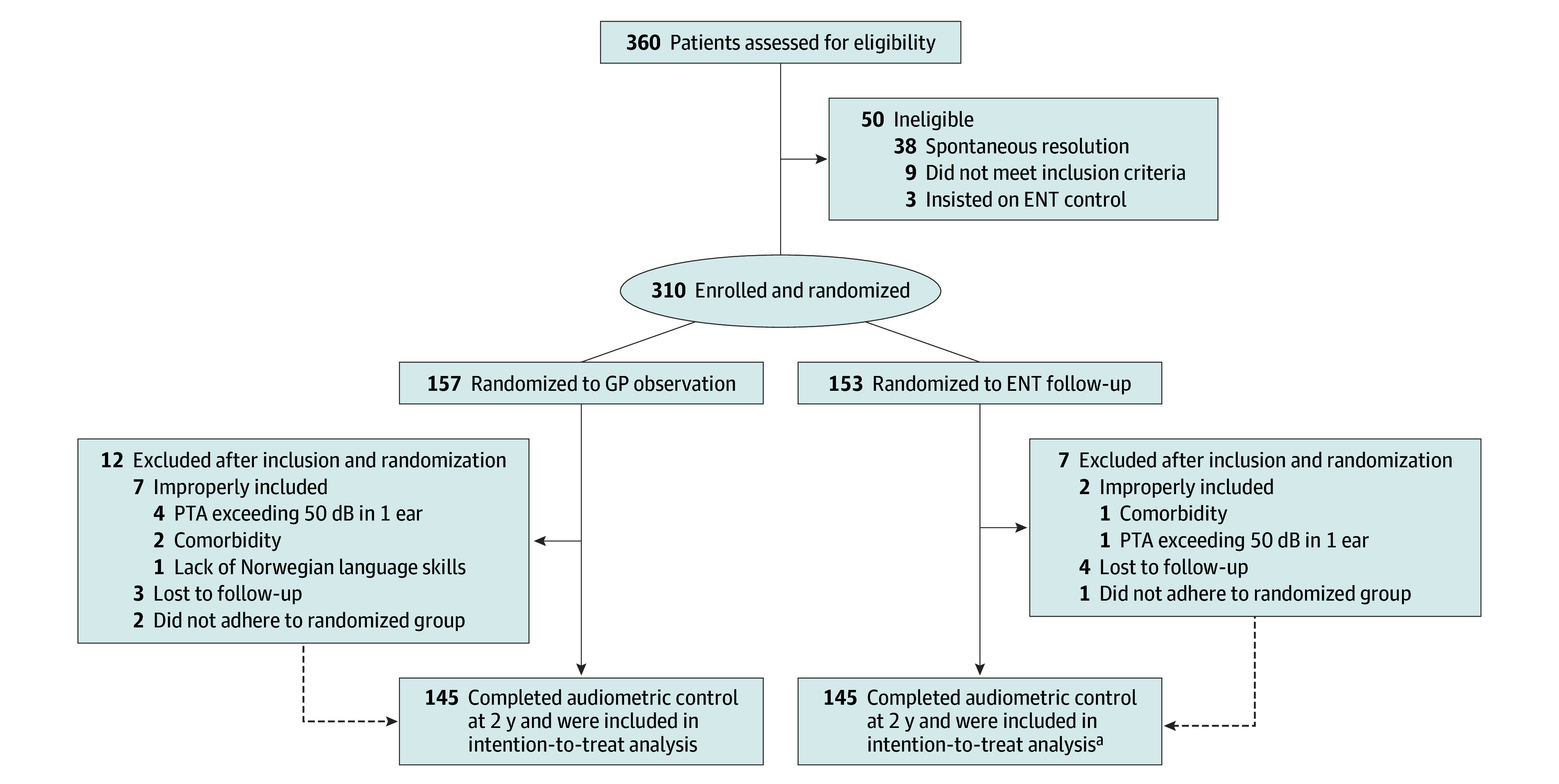
Trial Profile ENT indicates otorhinolaryngology (ear, nose, and throat); GP, general practitioner; PTA, pure-tone average. ^a^One additional patient did not complete audiometry but was present at the final control.

**Figure 2.**
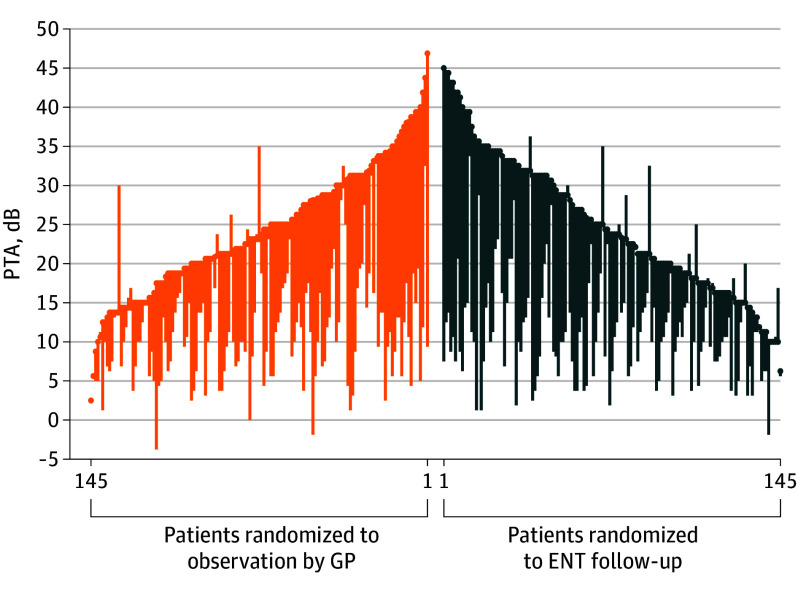
Comparative Outcomes Assessment for Primary Outcome Each point represents the PTA of a single patient at inclusion in dB. Each bar indicates the change in PTA from inclusion to control 2 years after ventilation tube insertion. ENT indicates otorhinolaryngology (ear, nose, and throat); GP, general practitioner; PTA, pure-tone average.

A total of 360 children were assessed for eligibility by participating clinicians through the ENT outpatient clinics of 6 hospitals in Norway (Figure 1). After VT insertion, 310 were randomly assigned to either observation by their GP with no routine follow-up or routine ENT follow-up (Figure 2). The main reason for exclusion was spontaneous resolution of OME. After randomization, 19 children were excluded; 3 due to comorbidities, 5 due to hearing loss exceeding exclusion cutoff, 2 chose follow-up through an ENT specialist in private practice, and 9 were lost to follow-up or withdrew from the trial. Baseline data were retained for use in LMM analysis, except for those excluded due to excessive hearing loss. Therefore, 305 patients (153 in GP arm and 152 in ENT arm) were included at baseline in the LMM analysis, while 290 patients (145 in GP arm and 145 in ENT arm) were included in the audiometric analyses. One patient in the ENT follow-up group underwent physical examination at control but did not complete audiometric control. Complication data from this patient were included for analysis.

Baseline demographics, preoperative audiometric data, and previous surgical history were well balanced between groups (Table 1). Of the 305 randomized patients, the median (IQR) age was 4 (3-6) years, 185 patients (60.7%) were male, and 120 (39.3%) were female. Tympanometry was taken on initial examination in 287 of 305 children (94%), with 229 of 287 (80%) being type B. Operative procedures performed at intervention time were also balanced. OME was listed as the indication for VT insertion for 270 individuals. Surgical indications were not mutually exclusive and, in some patients, adhesive or recurrent acute otitis media were cited as indications in addition to OME. Nearly all patients received Bobbin VTs (284 of 305 patients [94.4%]); however, 9 patients in the observation group and 8 in the ENT group received Donaldson VTs.

**Table 1.** Baseline Characteristics and Demographics of Randomized Patients

Characteristic	No. (%)		
	Observation (n = 153)	ENT (n = 152)	Total (N = 305)
Children demographics			
Age at inclusion, median (IQR), y	4.0 (4.0-6.0)	4.0 (3.0-6.0)	4.0 (3.0-6.0)
3-4	79 (51.6)	84 (55.3)	163 (53.4)
5-7	62 (40.5)	49 (32.2)	111 (36.4)
8-10	12 (7.8)	19 (12.5)	31 (10.2)
Sex			
Female	57 (37.3)	63 (41.4)	120 (39.3)
Male	96 (62.7)	89 (58.6)	185 (60.7)
Household			
Smoking household	4 (2.7)^a^	5 (3.3)	9 (3.0)
Guardianship status			
2-Parent household	137 (90.1)	127 (83.6)	264 (86.8)
Single-parent household	9 (5.9)	16 (10.5)	25 (8.2)
Shared custody	6 (3.9)	9 (5.9)	15 (4.9)
Parental demographics			
Parental age at inclusion, mean (SD), y	35.8 (5.3)	35.2 (5.9)	35.5 (5.6)
Highest level of parental education			
Lower secondary school	3 (2.0)	3 (2.0)	6 (2.0)
Upper secondary/vocational school	38 (25.0)	48 (31.6)	86 (28.3)
University for <4 y	48 (31.6)	50 (32.9)	98 (32.2)
University for ≥4 y	63 (41.4)	51 (33.6)	114 (37.5)
Employment status			
All caregiver(s) employed/student	138 (90.8)	138 (90.8)	276 (90.8)
1 of 2 Caregiver(s) employed/student	12 (7.9)	13 (8.6)	25 (8.2)
All caregiver(s) unemployed	2 (1.3)	1 (0.7)	3 (1.0)
Audiometric data			
Method of audiometry			
Pure tone	78 (51.0)	75 (49.3)	153 (50.2)
VRA	2 (1.3)	1 (0.7)	3 (1.0)
Play audiometry	73 (47.7)	76 (50.0)	149 (48.9)
PTA at inclusion, mean (SD)	24.7 (8.0)	25.6 (8.7)	25.1 (8.4)
Degree of hearing loss			
PTA ≤15	27 (17.6)	20 (13.2)	47 (15.4)
Slight (PTA 16-25)	63 (41.2)	66 (43.4)	129 (42.3)
Mild (PTA 26-40)	60 (39.2)	56 (36.8)	116 (38.0)
Moderate (PTA 40-50)	3 (2.0)	10 (6.6)	13 (4.3)
Tympanometric data			
Tympanometry performed, right side	142 (92.8)	143 (94.1)	285 (93.4)
Type B curve^b^	91 (64.1)	98 (68.5)	189 (66.3)
Type B curve with elevated volume^b^	3 (2.1)	3 (2.1)	6 (2.1)
Type C curve^b^	38 (26.8)	38 (26.6)	76 (26.7)
Tympanometry performed, left side	143 (93.5)	144 (94.7)	287 (94.1)
Type B curve^b^	98 (68.5)	95 (66.0)	193 (67.2)
Type B curve with elevated volume^b^	3 (2.1)	7 (4.9)	10 (3.5)
Type C curve^b^	35 (24.5)	38 (26.4)	73 (25.4)
No. of ears with type B curves at inclusion by patient			
None^b^	26 (18.2)	32 (22.2)	58 (20.2)
1^b^	45 (31.5)	31 (21.5)	76 (26.5)
2^b^	72 (50.3)	81 (56.2)	153 (53.3)
Surgical data^c^			
No. of participants	150	151	301
Surgical history^d^	54 (36.0)	51 (33.8)	105 (34.9)
VT insertion			
Once	34 (22.7)	34 (22.5)	68 (22.6)
Multiple	15 (10.0)	12 (7.9)	27 (8.97)
Adenoidectomy/adenotonsillectomy	30 (20.0)	21 (13.9)	51 (16.9)
Tonsillectomy alone	2 (1.3)	1 (0.66)	3 (1.0)
Surgical intervention at inclusion			
VT insertions, No.	150	151	301
Unilateral	40 (26.7)	26 (17.2)	66 (21.9)
Bilateral	110 (73.3)	125 (82.8)	235 (78.1)
Adenoidectomy/adenotonsillectomy	52 (34.7)	42 (27.8)	94 (31.2)
Tonsillectomy	0	1 (0.7)	1 (0.3)
Type of VT inserted			
Bobbin	141 (94)	143 (94.7)	284 (94.4)
Donaldson	9 (6.0)	8 (5.3)	17 (5.6)
Indication for VT insertion^e^			
OME	130 (86.7)	120 (79.5)	250 (83.1)
Recurrent purulent otitis media	13 (8.7)	16 (10.6)	27 (9.6)
Adhesive otitis	2 (1.3)	0 (0.0)	2 (0.7)
OME and recurrent purulent otitis media	4 (2.7)	12 (7.9)	16 (5.3)
OME and adhesive otitis	1 (0.7)	3 (2.0)	4 (1.3)

Abbreviations: ENT, otorhinolaryngology (ear, nose, and throat); OME, otitis media with effusion; PTA, pure-tone average; VRA, visual response audiometry; VT, ventilation tube.

^a^ Two patients in the observation group did not respond to the question pertaining to smoking habits.

^b^ Proportions represent participants in whom tympanometry was performed.

^c^ Proportions represent participants who underwent VT insertion.

^d^ Number of children who had previously undergone ENT surgery subdivided by intervention.

^e^ Indication for operation as reported by the operating physician. Indications were not mutually exclusive.

Two years after the insertion of VTs, patients allocated to the observation group had a mean PTA of 12.27 dB (95% CI, 11.07-13.48 dB]), while those allocated to ENT follow-up had a mean PTA of 12.44 dB (95% CI, 11.23-13.64 dB) (baseline mean PTA, 24.74; 95% CI, 23.77-25.71). LMM analysis showed PTA at 2-year control in the observation group to be noninferior to the ENT group (difference in means, 0.16 dB [1-sided 97.5% CI lower bound, −1.52 dB]). The lower limit of the 97.5% CI of the PTA difference between groups 2 years after VT insertion was well within the noninferiority threshold of 5 dB.

Retraction of the tympanic membrane was more prevalent in the ENT group (OR, 0.49 [1-sided 97.5% CI lower bound, 0.27]). Overall, there was no difference in the total number of complications between groups (OR, 0.67 [1-sided 97.5% CI lower bound, 0.39]; Table 2). There were no reported adverse events throughout the trial.

**Table 2.** Secondary Outcomes

Outcome	No. (%)			OR (1-sided 97.5% CI lower bound)
	Observation	ENT	Total	
Tympanometric data (per-protocol group), No.	145	145	290	NA
B curves at control^a^	21 (14.5)	34 (23.4)	55 (19.0)	0.55 (0.29)
Unilateral	19 (13.1)	20 (13.8)	39 (13.4)	0.85 (0.41)
Bilateral	2 (1.4)	14 (9.7)	16 (5.5)	0.13 (0.01)
Clinical control and complications, No.	145	146	291	NA
VT(s) present at control^b^	13 (9.2)	16 (12.4)	29 (10.7)	0.71 (0.30)
Unilateral	11 (7.7)	14 (10.9)	25 (9.2)	0.69 (0.27)
Bilateral	2 (1.4)	2 (1.6)	4 (1.5)	0.88 (0.06)
Reoperations^c^	18 (12.5)	30 (20.5)	48 (16.6)	0.54 (0.27)
Total No. of patients with complications	37 (25.5)	49 (34.0)	86 (30.2)	0.67 (0.39)
Recurrent OME	20 (13.8)	24 (16.6)	44 (15.2)	0.81 (0.40)
Unilateral	14 (9.7)	11 (7.6)	25 (8.6)	1.22 (0.49)
Bilateral	6 (4.1)	13 (9.0)	19 (6.6)	0.44 (0.13)
Perforation				
Unilateral	3 (2.1)	6 (4.2)	9 (3.1)	0.49 (0.08)
Retraction	23 (15.9)	40 (27.8)	63 (21.8)	0.49 (0.27)
Unilateral	14 (9.7)	25 (17.4)	39 (13.5)	0.47 (0.22)
Bilateral	9 (6.2)	15 (10.4)	24 (8.3)	0.51 (0.19)
Cholesteatoma	0	0	0	NA
Resolution				
Audiometric	128 (88.3) (n = 145)	123 (84.8) (n = 145)	NA	1.34 (0.68)
Tympanometric^d^	93 (88.6) (n = 105)	71 (75.5) (n = 94)	NA	2.62 (1.23)^e^
OME as indication for VT insertion^f^	Baseline, mean (95% CI) (n = 270)	2-y Follow-up observation, mean (95% CI) (n = 135)	2-y Follow-up ENT, mean (95% CI) (n = 135)	Difference in dB (1-sided 97.5% CI lower bound)
PTA, dB	25.26 (24.25 to 26.27)	12.35 (11.07 to 13.64)	12.53 (11.25 to 13.81)	0.17 (−1.62)

Abbreviations: ENT, otorhinolaryngology (ear, nose, and throat); NA, not applicable; OME, otitis media with effusion; OR, odds ratio; PTA, pure-tone average; VT, ventilation tube.

^a^ Number of patients with B curves at 2-year control. Ears with standing VT or perforation excluded from analysis.

^b^ Patients with standing VT who had been reoperated between inclusion and 2-year control were excluded from analysis.

^c^ Reinsertion of ventilation tubes within the 2-year follow-up period.

^d^ Participants with A curves at inclusion or standing VT with tympanometry in keeping with perforation were excluded from analysis (observation group [n = 105]; routine follow-up group [n = 94]).

^e^ Calculated as odds of nonresolution at 2-year control.

^f^ Analyses performed using a linear mixed model with time and treatment as fixed factors and identifier and unilateral VT insertion as random factors.

Subgroup analysis of PTA at 2-year control by sex, age, or PTA at inclusion, divided into tertiles, showed no difference between trial arms (eTable in Supplement 2). A total of 17 patients in the observation group and 22 patients in the ENT follow-up group had a PTA value of more than 20 dB at completion (OR, 1.34 [1-sided 97.5% CI lower bound, 0.68]). Tympanometric resolution, defined as the shift from a B curve to an A curve or C curve in at least 1 ear, was present in 93 of 105 participants in the GP follow-up group and 71 of 94 patients in the ENT follow-up group, yielding an OR of 2.62 (1-sided 97.5% CI lower bound, 1.23). An isolated analysis of the 270 patients with OME as indication for surgery also showed observation to be noninferior to routine ENT follow-up (0.17 [1-sided 97.5% CI lower bound, −1.62]).

## Discussion

In the multicenter ConVenTu noninferiority randomized clinical trial of the postoperative follow-up of children after VT insertion, observation without routine follow-up was found to be noninferior to routine ENT follow-up in terms of audiometric outcomes 2 years after insertion. Several indications were permitted for the insertion of VTs; however, audiometric findings were consistent when patients with OME as an indication for operation were analyzed in isolation.

Despite VT insertion being the most common ambulatory surgery in children,^1^ the follow-up of these patients has rarely been examined in clinical studies.^8^ A Cochrane study performed in November 2023 on VT insertion for OME^19^ did not assess level of care or strategy for subsequent follow-up, and both the AAO-HNS guidelines^2^ and NICE guidelines^10,11^ have recommended further research on this topic. The NICE guidelines published in August 2023 specifically state a lack of evidence surrounding a follow-up strategy for these patients and recommend a trial that nearly matches our protocol.^11^ An attempt to review evidence surrounding the postoperative follow-up strategy for patients with OME was performed for the NICE guidelines, which resulted in no articles meeting their criteria.^11^

We found no difference in the total number of complications between groups. There was, however, a higher prevalence of tympanic membrane retraction in the ENT follow-up group 2 years after surgery when counted as an isolated complication. Tympanometric resolution was lower in the ENT follow-up group when compared to the observation group. These results are difficult to explain. However, the lower rate of tympanometric resolution in the ENT follow-up group may be overrepresented due to the exclusion of patients with functional VTs present at control and those lacking B curves at inclusion.

This trial is, to our knowledge, the first sufficiently powered randomized clinical trial to examine observation as an appropriate alternative to ENT for the follow-up of children with VTs. Trial-specific GPs were not assigned to patients randomized to observation by their GP without routine follow-up, and thus received no special training as to the care for or examination of these patients. This variation in level of experience and physician-patient relationship was intended to reflect actual clinical practice. Including patients from multiple centers and not using trial-specific physicians makes our finding of noninferiority broadly generalizable.

The COVID-19 pandemic drastically reduced the number of VT surgeries. However, active effort put into reducing the number of patients who withdrew from the study resulted in a rate of 6.5% (20 of 310), slightly more than half of that accounted for in our originally powered study.

### Limitations

Of the limitations to this trial, the lack of audiometry at interim controls stands foremost. Audiometry is not routinely performed after VT insertion at all Norwegian institutions, and in a Swedish study with data taken from a Swedish registry that followed up patients with VTs, only 43% of patients underwent postoperative audiometry.^1^ Despite this, our results show noninferior hearing outcomes between groups at 2 years. One can, however, argue that PTA alone may be a suboptimal parameter to judge similarity between groups due to the expected amelioration of Eustachian tube function with age.^2^ This study also considered type C tympanograms to indicate the resolution of middle ear effusion. This is, however, not universally accepted and represents a limitation in this study.

The assessment of short-term complications, such as early tube extrusion, the development of granulation tissue, and tube occlusion, was not assessed in the observation group, as they were not scheduled for routine follow-up appointments. The justification for this was that treatment would not be indicated in asymptomatic patients, and guardians had been asked to contact their GP should their children develop symptoms. One could also postulate that results may have differed if these patients had been assessed on a regular basis.

Another limitation may be that no cholesteatomas were observed over the course of this study. While cholesteatoma is an important complication to report if present, it is both a rare^9^ and late^20^ complication which would not be expected in a study of this size and length of follow-up. An examination into the occurrence of cholesteatoma in these patients would require further research.

## Conclusions

The ConVenTu randomized clinical trial found that, 2 years after VT insertion, the mean hearing thresholds in otherwise healthy children between 3 and 10 years of age observed by a GP were noninferior to those who routinely followed up with ENT. Current guidelines suggest a follow-up regimen after VT surgery that requires extensive resources to maintain.^2,10^ With the growing importance of sustainability, finding a level of safe but sufficient care for patients with VTs can potentially liberate resources for the care of patients with complex and critical illnesses. The findings of this trial may help guide resource allocation for patients who undergo VT surgery.

## Notes

Supplement 1. Trial protocol and statistical analysis plan

Supplement 2. eAppendix. Information provided to general practitioners (GPs) and English translation eTable. Results of *t* testing of audiometric data and subgroup analysis

Supplement 3. Data Sharing Statement
