# Supplementary material for: Observation or Otolaryngology Surveillance After Ventilation Tube Insertion in Children: The ConVenTu Noninferiority Randomized Clinical Trial
Source: JAMA Otolaryngol Head Neck Surg. 2025 Oct 9;151(11):1063–70. doi: 10.1001/jamaoto.2025.2880 (PMC12512028; doi:10.1001/jamaoto.2025.2880)
Supplement: Supplement 1. — Trial protocol and statistical analysis plan [file jamaotolaryngolheadnecksurg-e252880-s001.pdf]

Protocol (Version 7) : Translated from the original Norwegian

-

Project Description:

The ConVenTu-study (Control of Ventilation Tubes)

- A randomized, controlled multicenter study -

Background

Ventilation tubes (drains) in the ear drum are one of the most common operations on children both in Norway and internationally (1,2). This intervention is performed to better hearing and speech development and to reduce ear complaints (3). Long term results of drain treatment are under discussion (4,5). A Cochrane report from 2010 concluded that drains had a positive effect on hearing in children with secretory otitis, but that effect was reduced after six to nine months (6). A systematic review of children with recurrent otitis concluded that drain insertion reduced the incidence of acute otitis with only one episode in the first six months after operation (7). Despite this, when this operation is performed follow up is planned to make sure that the drains are functioning, hearing is bettered, and potential complications are properly diagnosed and treated (8). Ecamples of complications are running ears, blocked tubes, early or lack of drain extrusion, persistent perforation of the ear drum or cholesteatoma (9).

According to national guidelines children are to be controlled until the middle ear is air filled and normalized, or the best possible result is attained, which can take several years. The first control is recommended after one month and thereafter every 4th month (10) while other countries have made follow up less comprehensive (11). A Scottish study found that few controls resulted in interventions and recommended that children be controlled after 3 months and thereafter only as needed (12). Swedens SBU (Statens beredning for mdicinsk utvardering) wrote in 2008 that ther is no documented assessments of how and when a child who has had implanted drains should be followed up (13).

In Norway children are primarily followed up in hospital or by Ear-Nose-Throat (ENT) doctors in private practice who have operated on them, with time intervals which vary from hospital to hospital. This often occurs less often than that which is recommended by guidelines. Many hospitals have problems completing these controls within the planned timeframe, due to long wait-lists and lack of capacity. Even though most hospitals still control all children who undergo drain operations themselves, St. Olavs Hospital implemented a change in 2007. From then controls of those children assumed to be the most healthy who received drains were delegated to their GP, while children with serious

hearing loss or other medical syndromes were controlled at hospital. GP controls were 6 and 18 months after the operation and guardians would have to order the control appointments for themselves. This follow up was evaluated through a retrospective study that did not find any difference in hearing, middle ear fluid or ear complaints 2 years after the operation (14). Another study on the same material showed that children came to GP controls even though they were not called inn, but many children were controlled by both GP and ENT regardless of where controls were originally planned (15).

There were, however, many limitations in this study. These included the small amount of material, the lack of randomization and that complications were not controlled over and above re-operations. In addition to this many GPs were unsure of how they should perform these controls (unpublished, own data). We would therefore like to perform a new and larger study to give more strength to recommendations nationally and internationally on the follow up of children after the implantation of drains. We will randomize children into two groups: ENT follow up and GP follow up as needed.

A potential benefit of follow up after intervention in hospital, may be the specialized equipment required for follow-up, while a potential benefit of GP controls may be short travel time and easier accessibility. As of now we know little about parents/guardians expectations to the intervention and follow-up as well as how this is experienced. We would therefore like to perform a qualitative part to this study.

Goals

The overall goal of the study was to find out if controls after drain implantation in the ear drum can be performed as needed by the GP without it having negative consequences for the patient. We also wish to examine if differing follow up after the drain implantation in the ear drum effects the long term effects of the operation.

The primary endpoints are the childrens' hearing two and four years after the operation and secondary endpoints are fluid in the middle ear, number of re-operations, subjective complaints, complication rate and patient satisfaction. Our nullhypothesis is that there is no difference in hearing and other effect variables, and the alternative hypothesis is that there is a difference.

The qualitative part of this study has, as a goal, to collect new information from parents/guardians to children between the ages of 3-10 years who receive implanted middle ear ventilation tubes. The goal is to gather knowledge about guardians expectations with drain treatment and follow-up. Semi-structured interviews of guardians from both follow-up groups will provide new and important information from users of health care services and contribute to overall knowledge base.

We wish to also undergo a health-economic analysis to calculate the course of the patients' treatment/follow up for patients in both the specialist and primary health care systems using data from the Norwegian patient register (NPR) and Control and payment of health refunds (KUHR) now Municipal patient and user register (KPR). This will provide insight into costs for differing levels of healthcare and knowledge about quality of life adjusted life years as a consequence of this intervention.

The results of this study are expected to be published in at least 3 articles which will form part of a PhD degree.

Consequences

The results of this study will contribute to giving increased knowledge on the follow/up of children after drain insertion in the ear drum. If the study should show that GP controls are defensible it will challenge todays situation of hospital based controls. That GPs perform controls will in this case give economic benefits. Alternatively, there may be consequences for the hospitals who already delegate controls to GPs if the study shows that children de not receive the follow up they require.

Materials and Methods

The parents of children are invited to participate in the study upon their outpatient appointment for assessment to drain insertion. They receive written information including the point of the study. The study additionally requires an audiological consultation with either an audiographer or doctor at the hospital before the operation as well as one at 2 and 4 years postoperatively. In addition to this they will be required to fill out questionnaires at all three timepoints. Self-reporting forms will contain amongst other things the validated OM 8-30 questionnaire which aims to broadly describe children's ear complaints and situation over the last 3 months (16). In addition to this quality of life will be recorded using The Pediatric Quality of Life (PedsQL) where we will use the parent reported forms for children dependent on how old they are (forms can be found for 2-4 year olds, 5-7 year olds and 8-12 year olds)(17, 18) and the Strengths and Difficulties Questionnaire's Norwegian version (SDQ-Nor)(19). Previous audiological information will be collected from the journal system for example preoperative hearing and dates for previous drain insertions.

In the qualitative part of this study an individual semi-structured interview will be conducted of a choice of guardians from both follow-up groups from up-to 3 timepoints; within the first 14 days, 6 and 24 months after drain insertion.

Data from NPR and KUHR/KPR will be collected for the health economic analyses (see examinations and measurements page 7)

Design and procedure

Randomized multicenter study where the primary goal is childrens change in hearing. Other audiological data, number of reoperations, subjective problems, complication rate and patient satisfaction will also be examined. We will follow the CONSORT rules. Questions regarding enrollment in the study as well as consent occur at the outpatient clinic prior to operation for children who are planned in to operation. Inclusion and randomization occur immediately after the child's operation. This, to prevent the inclusion of patients who would later be stricken from the operating list. Data collected from those who are not operated on will be shredded.

Children will be randomized to follow up after operation to either the hospital or to the GP. Follow-up through the hospital involves a control every 6 months as long as there is a clinical need for it. GP controls will only be performed as required. Guardians will be given written information as to when drain controls are recommended through their GPs; for example with ear secretion, persistent reduced hearing, ear infection, etc. GPs will receive a procedure for controls and how one should tackle the most common complications. This procedure will be attached to the discharge report from drain insertion so that it will be available for the GP under the consultation.

Randomization

Stratified sampling with "center" as stratum (balances within each center) and block randomization will be used to assure like distribution at almost all timepoints. One applys for help of the department for applied clinical research, DMF, NTNU, for help with the practical part surrounding completion of randomization as well as the computer program ("WebCRF") which is used for registration and randomization.

Inclusion Criteria

Children 3-10 years of age who receive an implanted drain through St. Olavs Hospital, Molde Hospital, Ålesund Hospital, Kristiansund Hospital, Gjøvik Hospital Innlandet and Stavanger Universityhospital. The lower age bracket is set to allow for hearing tests of children. The upper age bracket is set as children older than 10 years of age at initial operation often have additional illnesses which would prevent them from being candidates in this study.

Exclusion Criteria

Medical syndroms or other serious comorbidities which give an expected higher complication rate after drain insertion, such as Downs syndrome, cystic fibrosis or primary ciliary dysfunction. Diagnosed auditory processing disorder (APD). Known, serious

sensorineural hearing loss in at least one ear (> 50dB hearing threshold in one frequency between 0,25 and 4,0 kHz).

Parents of children who do not master Norwegian both written and spoken.

Number of participants

The calculation of the number of participants was performed by the statistician. The number depends on which factors which are to be analyzed and which statistical method will be used. We are interested in determining differences in effect of treatment in disfavor of the GP-group, but a better result in this group would be optimal in regards to the clinical implications (performing therefore a two sided test. No difference between groups is also a possibility and of interest (equivalence study). With a focus on the likeness between groups it is especially important to avoid falsely concluding "no effect" (type II error), which implies that we want a high power for the test for difference between groups, minimum 90%, preferably up to 95%.

The primary end-point is hearing, which is measured in dB (continuous variable). Wi examine changes in average hearing in the frequencies 0,25-4,0 kHz between 2 timepoints. The smallest clinically relevant change in hearing is 5dB. With an expected bettering of 10dB in the one group and 5dB in the other group (absolute diff. 5dB), and estimated standard deviation of 10dB in each group, we would with a significance of 5% need 85 (105) participants in each group to determine a difference with 90% (95%) power. To account for skew from the normal distribution, at least 15% should be added (increase to 98 or 131 in each group) and an additional 20% for drop-outs. A minimum of at least 118(157) participants in each group would be required. To maintain power in a model where we account for study center (random factor), and other clinical or demographic factors, the number must be increased (by at least 10 cases per variable. The total is now increased from 314 to 400, 200 participants in each group due to multiple participating hospitals.

Analysis of categorical variables require generally speaking a larger data material to reach a similar strength to continuous, normally distributed variables. The incidence of fluid in the middle ear (categorical variable) is expected to come in 80-90% of patients prior to operation. Given an expected amelioration of 90% in the one group and 85% in the other group (absolute difference in proportions of 0.05), we need to include at least 920 (1110) patients in each group to reach a strength of 90% (95%) for this statistic test. With a strength of 80%, which is acceptable to find a difference between groups (and less focus on mistakenly keeping the null hypothesis), the number would be reduced to 680. This number will be hard to achieve within the boundaries set for this project. A larger

statistically significant difference between groups would still be possible to find (generalizable to the population).

Around 800 children per year are operated on at the six participating hospitals together. We assume that around 600 of these are actual for our study, the rest will most likely be excluded due to comorbidity or age. Some will reject inclusion in the study. In a timeframe of 1-2.5 years for the inclusion of patients, and in economical bounds, it is realistic to include a maximum of 400 participants in this study, 200 randomized to each group. For categorical variables this is acceptable despite not having increased participants per study center because we assume that there are little demographic differences between study centers.

In the qualitative part of the study a choice of guardians of included children in the ConVenTu-study will be invited. The guardians will be recruited from multiple, but not nescessarily all, participating hospitals (St Olavs Hospital and hospitals in Kristiansund, Molde, Ålesund, Innlandet Gjøvik and Stavanger). We will include guardians to children from both follow up groups, and with differing ages, sexes and socioeconomic backgrounds. The inclusion will occur until desaturation is achieved. We expect the amount to not exceed 20 guardians from each follow up group. Participants are informed both orally and written and a separate consent will be signed should they want to participate. Participants will not be required to bind themselves to all three interviews at the first interview.

<u>Preoperative examinations and measurements</u>

1. Self report form where audiograph and other health care personell at the audiological center/outpatient clinic enter the guardians' answers into a database («Web-CRF»). Information about ear complaints, subjective hearing and sociodemographic background questions, operation form and questionnaires regarding quality of life "OM 8-30", "PedsQL" and "SDQ".
2. Hearing testing, including puretone audiometry (0.25-4.0 kHz) (20) and speech audiometry (21). Should the child due to age or othe reasons be unable to cooperate with these tests, other test methods may be used, for example TEOAE (transient otoacustic emissions)(22). Speech audiometry is performed using child lists, one syllable words, two syllable words or three word utterances or speed test three word utterances (23).
3. Tympanometry (pressure measurement)(24).

<u>Examinations and measurements 2 and 4 years after operation</u>

1. Self report form where audiograph and other health care personell at the audiological center/outpatient clinic enter the guardians' answers into a database («Web-CRF»). Information about ear complaints, subjective hearing and sociodemographic background questions, operation form and questionnaires regarding quality of life "OM 8-30", "PedsQL" and "SDQ".
2. Audiolgical testing as before operation.
3. Journal examination in regards to complications and new drain operations.
4. If required the GPs journal may be requested.

Health Economic Examinations after 2 and 4 year follow ups

1. Data from NPR and KUHR/KPR:

Date of treatment, diagnosis and procedure codes (ICD-10 and NCSP)

Type and length of treatment (day, full day or outpatient)

Costs (DRG-code, DRG-weight and corrected DRG-weight)

Level of care (hospital, GP, GP acute and emergent)

Reimbursement code and information regarding patient payment (HELFO)

In the qualitative part of this study an individual semi-structured interview will be conducted of a choice of guardians from both follow-up groups from up-to 3 timepoints; within the first 14 days, 6 and 24 months after drain insertion. The interview guide will at each timepoint have neutral and open questions about themes that contain the guardians' expectations and experiences related to the operation or follow up after drain operation. Themes which spontaneously come up under the interview will be explored further.

All interviews are planned to be completed by the same researcher with a length not normally exceeding 60 minutes. Interviews will be performed with the help of recording and transcribed verbatim. Interviews will be performed such that personal information is protected. The original interviews are planned to be completed in person at the institution near the patient for example their associated hospital or by home visit. The Covid-19 pandemic required the use of telephones for these interviews.

Statistical analysis

Linear mixed model (LMM) with center as random factor will be used to examine if there is a significant long-term effect (at 2 and 4 years) of treatment (insertion of drain) on hearing level and other audiological and clinical factors. Interaction joints will be included in the statistical model to examine the effect of treatment, measured in the form of change compared to pre-operative measurements, is the difference between patients who receive

follow-up through their GP or hospital. Log transformation of data will be actual if it is relevant to study relative over absolute changes, and transformation of data will be actual if a response variable appears to be skewed. Alternative non-parametric models can be used as needed.

Differences between the two randomization groups (hospital, GP) taking into account severity of complications, number of reoperations and proportion of re-referrals under the follow up period will be examined with the help of chi-squared tests (as a categorical variable) or Mann-Whitney test of differences of the entirety. It will also be actual to compare groups taking into account re-operation rate, and identify riskfactors for recurrence (re-operation), by using different types of survival analysis models, alternatively a logistic regression (if one can not account for time for re-operation).

If there is no significant difference between groups, we will focus on magnitude of differences between groups. With a lack of significant difference we will use a 95% confidence interval for absolute difference around 0 (around 1 for relative difference), but border values in the interval must be less than that which is considered a clinical difference of significance. What these values are will depend on which factors are being analyzed.

The health economic analyses will be performed based on randomization and where follow up has been performed.

In the qualitative portion of the study the transcription of interviews will be done continuously. Multiple researchers belonging to the research group and the project will collaborate to read and analyze the transcript. The analysis method will be adjusted to the data and presented problems.

Ethics

Studies of drain insertion in children have not previously found a difference in hearing function with GP follow up when compared to hospital follow up (14, 15). Children where one expects an increased complication rate after drain insertion will be excluded from the study and will be followed at the hospital. Should complications of worsening occur the GP will refer the child back to the nearest hospital in keeping with other children who require help in the specialist health care system. In addition to this they will receive a direct number to ENT which the GP can use should they have questions regarding the participants, and they will as need be provided with a quick consultation. All participants will be controlled by an ENT doctor or audiographer before the operation, as well as after 2 and 4 years. The children who, due to registered findings, have a need for further follow up or consultation, will be offered so quickly.

There is normally no risk associated with audiometry, tympanometry or the other tests which are planned to be used in this study. This is normal treatment procedure in the examination of hearing and ear drums in the specialist health care system.

Informed consent will be collected surrounding participation in the study. Guardians must consent for themselves and by proxy for their children. In the information brochure about the study it is also stated that children have the ability to refuse and that it is possible to drop out of the study after having consented. Those that require additional information are asked to take contact by telephone. Project participants are included in the study only after having turned in written consent. Consultations related to this study are free of charge. Those who do not wish to participate will be followed up in hospital as is normal.

Indirect identifiable information from NPR and KUHR/KPR will give a significant supply of knowledge for the study, but will also be an extra burden for the participants. The attachment of treatment information was not requested in the initial REC-application and existing information and consent does not cover the attachment of such data. A new information and consent form is therefore being made which will be provided to further inclusions. An additional consent and information form will be provided to those already included in the study. For those who have completed 2 year follow up a new consent sheet will be mailed to them. Those who have not yet completed 2 year control will be asked for additional consent at the 2 year control. Refusal will not bear consequences for their access to a 2 year control as planned.

In the qualitative part of the study written information handout and consent form will be provided to the guardians before participation after their child is included in the main study. Benefits to participation in the qualitative study are that the guardians can share their own expectations and experiences from their child’s treatment and follow up. This takes care of the user perspective. We do not consider the time used to interview to be a problem. Refusal will not bear consequences for participation in the main study.

Privacy

Informants (children, medical data and parent answers on self report forms) will not be identifiable without the use of a special ID number. Electronic data and that which the researchers will use will only have the ID number and intials under the analysis of data. Project assistents will know the patients identity and key to the ID number while data is being collected. The key as well as the data itself will be kept in a locked area outside of the use of project assistants. Audiometric data will be saved in the child’s hospital journal since this is assumed to be important information for the patients regardless of this study.

One applys for help of the department for applied clinical research, DMF, NTNU, for the use of “WebCRF” a solution with good data security. Registered data is secured also when they are deidentified and registered. PC-access is orotected by username and password. The project leaders, researchers and coordinators PCs are networked.

The project is approved by the Regional committee for medical research ethics in south east B (2015/902).

In the qualitative part of the study sound files will be deleted as soon as the interviews are transcribed and controlled. The transcribed interviews will not contain indentifiable data. Participants will be referred to as informant 1,2,3, etc. The ID key from the main study exists, is physical and is lock up at St Olavs Hospital. Transcribed interviews are saved electronically on access controlled file areas on St. Olavs hospital servers.

The qualitative study is previously treated as a quality assurance project (previously assessed by the REK, then as a separate project, attached to ConVenTu) and approved by St Olavs Hospital. Since the qualitative part of the study would like to include different regions and will be performed by the same people as ConVenTu and therefore wishes to be included as a completely integrated study under ConVenTu a change request has been filed. The privacy representatives believe this to be useful from a privacy perspective.

ID number:

Each patient will be coded with a number which is attached to a name and government personal ID number when the patient is included in the study and is registered in “Web-CRF”. This coding will be performed at the audiology center/out-patient clinic at each respective hospital on the specially designed form. The same ID number will be used on all information regarding the child and its parents. Personal information will be stored physically locked in the project office at each hospital as long as the study is being performed and will be shredded at most 5 years after project completion 31.12.2032. The registration of data available to the researcher is not personally identifiable.

Timeline:

2014 fall: Planning, design, inclusion of collaborators.

2015: Application to REK, register in Clinical Trials, Apply for funding, Hire Project coordinator, project assistants and audiographers.

2017 fall: Begin data collection. Estimated time 2.5 years.

2019 fall: Begin 2-year controls after operation, estimated time 2.5 years.

2020 fall: Begin qualitative study, own institution.

2021 spring: Begin qualitative study in different parts of Norway.

2022 spring: Hire PhD stipendiat when 2-year data is finished being collected.

2022-2025: Analysis and article writing.

2021 fall: Begin 4-year control after operation, estimated time 2.5 years (finished spring 2024)

2024: Hire new post-doc/stipendiat as needed.

2027: Planned end to study.

Research group

Responsible Institution:

The medical faculty of the Norwegian institute of science and technology: Institute for public health (ISM) and the institute for neuromedicine and movement (INB). The project was performed in collaboration with the ENT-departments of St Olavs Hospital, Ålesund hospital, Molde hospital, Kristiansund hospital, Gjøvik hospital Innlandet and Stavanger University hospital.

Applicants:

Anne-Sofie Helvik, Professor at ISM, NTNU and research consultant for ENT, St Olavs Hospital. Bjarne Austad, PhD. specialist i family medicine ved Øya Legesenter. Wenche Moe Thorstensen, PhD. 1.amanuensis Institute for Neuromedisin (INM), NTNU and ENT doctor, St Olavs Hospital. Ann Helen Nilsen, PhD, research nurse at ENT department, St Olavs Hospital.

Responsible Research Institutions:

St Olavs Hospital: Marit Fagerli, Clinic head for ENT, Maxillofacial surgery and Ophthalmology, post.onh.kjeve.oye@stolav.no

Kristiansund, Molde og Ålesund Hospital: Ingrid Vasdal, Clinic head ENT, postmottak@helse-mr.no

Stavanger University hospital: Research director Svein Skeie, post@sus.no

Gjøvik Hospital, Innlandet: Research director Ingeborg Hartz, forskning@sykehuset-innlandet.no

Project collaborators:

St Olavs Hospital: Department head Ståle Nordgård

Kristiansand Hospital: Department head Mikal Gjellan

Ålesund Hospital: Department head Lars Friedrich Duwe, ENT doctor Sverre Zahl.

Molde Hospital: ENT doctor Espen Homleid Alseth

Gjøvik Hospital, Innlandet: Department head Geir Sæthermoen, ENT doctor Paul Olav Stadtler

Statistician: Professor Grethe Albrektsen, Department for applied clinical research, NTNU

References:

1. Rosenfeld RM, Schwartz SR, Pynnonen MA, Tunkel DE, Hussey HM, Fichera JS, et al. Clinical practice guideline: tympanostomy tubes in children. Otolaryngol Head Neck Surg. 2013;149(1 Suppl):S1-S35.

2. Kvaerner KJ, Kristiansen HA, Russell MB. Otitis media history, surgery and allergy in 60-year perspective: a population-based study. Int J Pediatr Otorhinolaryngol. 2010;74(12):1356-60.

3. National Institute for Health and Care Excellence (NICE). Surgical Management of Otitis Media with Effusion in Children: National Collaborating Centre for Women's and Children's Health (UK); London: RCOG Press; 2008 2008 Feb.

4. Caye-Thomasen P, Stangerup SE, Jorgensen G, Drozdziewic D, Bonding P, Tos M. Myringotomy versus ventilation tubes in secretory otitis media: eardrum pathology, hearing, and eustachian tube function 25 years after treatment. Otology & neurotology : official publication of the American Otological Society, American Neurotology Society [and] European Academy of Otology and Neurotology. 2008;29(5):649-57.

5. Paradise JL, Feldman HM, Campbell TF, Dollaghan CA, Rockette HE, Pitcairn DL, et al. Tympanostomy tubes and developmental outcomes at 9 to 11 years of age. N Engl J Med. 2007;356(3):248-61.

6. Browning GG, Rovers MM, Williamson I, Lous J, Burton MJ. Grommets (ventilation tubes) for hearing loss associated with otitis media with effusion in children. Cochrane Database Syst Rev. 2010(10):CD001801.

7. Lous J, Ryborg CT, Thomsen JL. A systematic review of the effect of tympanostomy tubes in children with recurrent acute otitis media. Int J Pediatr Otorhinolaryngol. 2011;75(9):1058-61.

8. Isaacson G. Overview of tympanostomy tube placement and medical care of children with tympanostomy tubes UpToDate2013 [updated May 9th 2012; cited 2013 June 3rd].

Available from: http://www.uptodate.com/contents/overview-of-tympanostomy-tube-placement-and-medical-care-of-children-with-tympanostomy-tubes?source=see_link&anchor=H24#H24.

9. Kay DJ, Nelson M, Rosenfeld RM. Meta-analysis of tympanostomy tube sequelae. Otolaryngol Head Neck Surg. 2001;124(4):374-80.

10. Norwegian Society of Otorhinolaryngology HaNS. Norwegian Society of Otorhinolaryngology and Head & Neck Surgery [http://legeforeningen.no/Fagmed/Norsk-Forening-for-Otorhinolaryngologi-Hode--og-Halskirurgi/Veileder-for-ore-nese-halsfaget/Otologi/Sekretorisk-otitt/] (National recommended guidelines) 2011 [cited 2012 June 20th].

11. Derkay CS, Carron JD, Wiatrak BJ, Choi SS, Jones JE. Postsurgical follow-up of children with tympanostomy tubes: results of the American Academy of Otolaryngology-Head and Neck Surgery Pediatric Otolaryngology Committee National Survey. Otolaryngol Head Neck Surg. 2000;122(3):313-8.

12. Spielmann PM, McKee H, Adamson RM, Thiel G, Schenk D, Hussain SS. Follow up after middle-ear ventilation tube insertion: what is needed and when? J Laryngol Otol. 2008;122(6):580-3.

13. The Swedish Council on Technology Assessment in Health Care. Rörbehandling vid inflammastion i mellanöret [Tympanostomy tube insertion for otitis media in children] (in Swedish, English translation). En systematisk litteraturöversikt. Stockholm: SBU; 2008. p. 78-84.

14. Austad B, Hetlevik I, Bugten V, Wennberg S, Olsen AH, Helvik AS. Can general practitioners do the follow-ups after surgery with ventilation tubes in the tympanic membrane? Two years audiological data. BMC Ear Nose Throat Disord. 2014;14(1):2.

15. Austad B, Hetlevik I, Bugten V, Wennberg S, Olsen AH, Helvik AS. Implementing guidelines for follow-up after surgery with ventilation tube in the tympanic membrane in Norway: a retrospective study. BMC Ear Nose Throat Disord. 2013;13:2.

16. Dakin H, Petrou S, Haggard M, Benge S, Williamson I. Mapping analyses to estimate health utilities based on responses to the OM8-30 Otitis Media Questionnaire. Quality of life research : an international journal of quality of life aspects of treatment, care and rehabilitation. 2010;19(1):65-80.

17. Reinfjell TJ, T. Måleegenskaper ved den norske versjonen av The Pediatric Quality of Life Inventory 4.0 (PedsQL). PsykTestBARN. 2012;2(1).

18. Reinfjell T, Hjemdal O, Aune T, Vikan A, Diseth TH. The Pediatric Quality of Life Inventory (PedsQL) 4.0 as an assessment measure for depressive symptoms: a correlational study with young adolescents. Nordic journal of psychiatry. 2008;62(4):279-86.

19. Eilertsen ME, Rannestad T, Indredavik MS, Vik T. Psychosocial health in children and adolescents surviving cancer. Scand J Caring Sci. 2011;25(4):725-34.

20. 8253-2 I. ISO 8253-2:2009. Acoustics - audiometric test methods. Part 2: Sound field audiometry with pure tone and narrow-band test signals. : Geneva: International Organisation for Standardization; 2009. Available from: http://www.iso.org/iso/home/store/catalogue_tc/catalogue_detail.htm?csnumber=51997.

21. 8253-3 I. ISO 8253-3:2012. Acoustics -- Audiometric test methods -- Part 3: Speech audiometry Geneva: International Organisation for Standardization. Available from: http://www.iso.org/iso/home/store/catalogue_tc/catalogue_detail.htm?csnumber=45101.

22. Boone RT, Bower CM, Martin PF. Failed newborn hearing screens as presentation for otitis media with effusion in the newborn population. Int J Pediatr Otorhinolaryngol. 2005;69(3):393-7.

23. Øygarden J. Norwegian speech audiometry. Trondheim: Norwegian University of Science and Technology; 2009.

24. Onusko E. Tympanometry. Am Fam Physician. 2004;70(9):1713-20.

Table of Contents

1. Statistical Analysis Plan **2**
2. Signature page **3**
3. Abbreviations **4**
4. Introduction **5**
4.1 Background and rationale 5
4.2 Trial objectives 5
5. Trial methods **5**
5.1 Trial design 5
5.2 Randomization 5
5.3 Sample size 5
5.4 Statistical framework 6
5.4.1 Hypothesis test *6*
5.4.2 Statistical interim analyses and stopping guidance *6*
5.4.3 Timing of final analysis *6*
5.4.4 Timing of the outcome assessments *6*
5.5 Blinding procedure 7
6. Statistical principles **7**
6.1 Confidence intervals and p-values 7
6.2 Adherence and protocol deviations 7
6.3 Analysis groups 7
7. Trial sample **7**
7.1 Screening data, eligibility and recruitment 7
7.2 Withdrawal/Follow-up 8
7.3 Baseline patient characteristics 8
8. Analysis **8**
8.1 Analysis of the primary efficacy endpoint 8
8.1.1 Pure tone average (PTA) *8*
8.1.2 Statistical analysis *8*
8.1.3 Missing data *8*
8.2 Subgroup analyses 9
9. Safety analyses **9**
10. Statistical software **9**
11. References **9**

1. Statistical Analysis Plan

The Statistical Analysis Plan of October 31st 2023 has not been changed and is final:
Adminitrative information:

Sponsor name	The Institute of Neuromedicine and Movement Science (INB), Norwegian University of Science and Technology
Sponsor address	Nevro Øst, Edvard Griegs gate 8, 7030 Trondheim
REC no.	2015/902
Trial title	Postoperative Controls of Ventilation Tubes in Children - by General Practitioner or Otolaryngologist? A Multicenter Randomized Controlled Trial (The ConVenTu Study)
Trial registration no.	NCT02831985

SAP and protocol version

SAP version and date	This SAP is version 1, dated October 31st 2023
Protocol version	This document was written based on the information contained in the study protocol[1] and the clinical trials website as of 31.10.2023.

SAP revision history

Protocol version	SAP version	Section number changed	Description and reason for change	Date changed
1.0	1.0	NA	First edition of SAP	October 31st 2023

2. Signature page

Author :
Resident in otorhinolaryngology and PhD candidate Rikki Kyle Kotyk Yahiro, MD
Department of Otolaryngology, Head and Neck surgery
St Olavs Hospital
7006 Trondheim, Norway

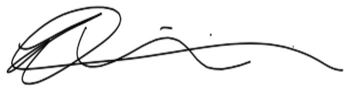

Signature ______________________ 31.10.2023 Date (dd/mm/yyyy)

Principal/coordinating investigator :
Professor, Consultant otorhinolaryngologist Wenche Moe Thorstensen, MD PhD
Department of Otolaryngology, Head and Neck Surgery
St. Olavs Hospital
7006 Trondheim, Norway

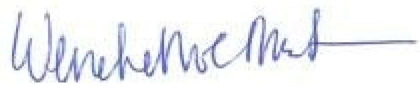

Signature ______________________ 31.10.2023 Date (dd/mm/yyyy)

Trial statistician :
Associate professor Øyvind O. Salvesen, MSc PhD
Norwegian University of Science and Technology
7006 Trondheim, Norway

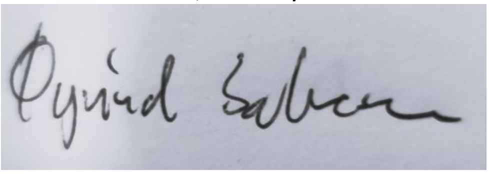

Signature ______________________ 31.10.2023 Date (dd/mm/yyyy)

Study Director :
Professor Anne-S. Helvik, PhD
Department of Nursing and Public health
Norwegian University of Science and Technology
7006 Trondheim, Norway

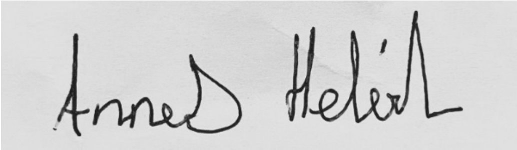

Signature ______________________ 31.10.2023 Date (dd/mm/yyyy)

3. Abbreviations

PTA	Pure tone average
VT	Ventilation tube
GP	General practitioner
HRQoL	Health-related quality of life
SD	Standard deviation
ENT	Ear, Nose and Throat
LMM	Linear Mixed Models

4. Introduction

4.1 Background and rationale

Otitis media with effusion is a major cause of acquired hearing problems in children[2]. Operative treatment with the implantation of ventilation tubes (VTs), also called tympanostomy tubes or grommets, is indicated in cases of persisting or recurrent otitis media[3]. This is the most common ambulatory surgery performed on children[3], and in Norway approximately 6700 children undergo surgery annually[4]. Post-operative controls are predominantly carried out by otolaryngologists within the specialist health care system[3,5] and may continue for two or more years[6]. These controls are performed to assess whether the tubes are functional, whether hearing loss has improved and to properly treat potential complications[3,5,7]. A meta-analysis performed in 2001 concluded that sequelae after VTs are most often transient or cosmetic in nature[7]. VT removal is recommended after 2 years if they have not been spontaneously expelled[3,5,6,8]. This is done to reduce the risk for a persistent perforation of the tympanic membrane[3,5,6,8].

The ConVenTu study is a multicenter randomized non-inferiority study which compares the hearing of patients treated with tympanostomy tubes where controls were performed by a general practitioner (GP), to those controlled within the specialist health care system. The aim is to determine whether controls performed by the GP are a safe and sufficient alternative to those controlled within the specialist health care system.

4.2 Trial objectives

The primary objective of this study is to determine whether there is a difference in middle ear function in patients where post-operative controls were performed by the GP compared to those controlled within the specialist health care system.

5. Trial methods

5.1 Trial design

This is a multicenter randomized non-inferiority study involving six centers in Norway and organized through St. Olavs Hospital, Trondheim, Norway. Patients were included through Gjøvik hospital, Stavanger University Hospital, hospitals in Ålesund and Molde as well as a private practice in Kristiansund. An initial assessment of eligibility was performed through the out-patient ENT clinic upon further referral for operative implantation of tympanostomy tube(s). The surgical procedure was performed at one of the aforementioned hospitals.

5.2 Randomization

The randomization procedure is computerized and carried out utilizing the "WebCRF" software developed at the Unit for Applied Clinical Research at The Faculty of Medicine and Health Sciences, NTNU[9]. The design is block randomization with blocks of varying sizes, with an allocation ratio of 1:1, stratified on the study center.

5.3 Sample size

The sample size was calculated prior to inclusion by a statistician. Hearing thresholds are determined in 5 dB steps and equality in PTA 2 years after surgery (primary endpoint) was

therefore defined as < 5 dB (5 dB as equivalence margin). To avoid an incorrect conclusion of 'no difference' between the groups (type II error), the power of the tests for detecting a difference of ≥ 5 dB must be high (≥ 95%, error margin < 5%). Thus, with a power of 95% and a significance level of 5% (two-sided test), 105 participants are needed in each group to be able to detect an absolute difference in mean PTA between the groups of ≥ 5 dB (SD 10 dB) in each group based on the results of our previous study[10,11]. 15% was added to mitigate a potential skew distribution of hearing level, and an additional 20% to account for potential dropouts, we are left with a sample size of 145 participants in each group. To maintain power in an analysis stratified for six study centers (five additional parameters in model), the total sample size must be increased by at least 50 participants, applying the general rule of thumb of at least 10 cases per parameter. If we assume that the 50 participants are evenly distributed in the two randomization groups, this leaves us with a sample size of at least 170 participants in each group. We initially planned to truncate up to 200 participants in each group to maintain power in case of a higher dropout rate. However, during the pandemic the surgery rate in Norway dropped dramatically, and with it our inclusion rate. After a preliminary assessment of our drop-out rate and recalculation by the project's statistician, it was determined that 135 participants in each group would be sufficient.

5.4 Statistical framework

5.4.1 Hypothesis test

Primary outcomes will be determined by measuring the change in the average of measured frequencies between 500Hz and 4kHz between baseline and control 2 years after the operative implantation of tympanostomy tubes. The null hypothesis is that controls performed by the GP lead to poorer PTA results 2 years after implantation of tympanostomy tubes compared to those performed within the specialist health care system. The alternative hypothesis will be that the mean change in PTA of patients controlled by their GP is not inferior to the PTA of patients controlled in the specialist health care system.

The test will be performed at the two-sided 5% significance level. A difference between the two groups will be claimed if the null hypothesis is rejected (p-value less than 5%). Superiority of controls within the specialist health care system will be claimed if the two-sided p-value in the test comparing the change from baseline is less than 5% and if the effect goes in favor of controls performed within the specialist health care system.

5.4.2 Statistical interim analyses and stopping guidance

There will be no interim analyses performed on this trial.

5.4.3 Timing of final analysis

The main analysis is planned when all participants have been to a clinical control 2 years following implantation of ventilation tubes.

5.4.4 Timing of the outcome assessments

Clinical assessment and inclusion. Initial evaluation of eligibility.	Day 0
Collection of informed consent to study.	Day 0

Operative treatment: Installation of tympanostomy tubes.	-
Clinical control and collection of control PROM forms.	Op. + 2 years.

5.5 Blinding procedure

Due to the nature of the procedure the patient and surgeon were not blinded. Patients were randomized after inclusion and further follow-up instructions given on the day of the operation. Follow-up controls were either routine through the hospital or as requested by the patients' GP and therefore not blinded. The audiographers responsible for testing were blinded to the patients' allocated group. The data collected will be divided into two groups within our database and assigned a number according to controls through the GP or specialist health care system by a third party. These numbers will be unknown to the outcomes assessor until completion of data analysis. Results from the questionnaires regarding controls performed by the GP or within the specialist health care system will be omitted from the primary analysis to prevent unblinding.

6. Statistical principles

6.1 Confidence intervals and p-values

All calculated p-values will be 2 sided and compared to a 5% significance level. If a p-value is less than 0.05, the null hypothesis will be discarded. Efficacy estimates for the two alternatives will be presented with two-sided 95% confidence intervals.

6.2 Adherence and protocol deviations

Upon drafting of the protocol a seventh hospital was to be included. Due to problems involving the inclusion of patients they removed themselves from the study without contributing to the data set. In addition to this our initial goal was to include 400 patients. However, due to a drastic reduction in the implantation of tympanostomy tubes under the Covid-19 pandemic this was reassessed, and that number was reduced to 135 patients per group. Cases which do not adhere to the study protocol will be excluded.

6.3 Analysis groups

- All randomized patients (All randomized patients, including those lost to follow-up.)
- Full analysis set (All patients which were randomized and treated.)
- Complete case set (All data present at both baseline and control)

7. Trial sample

7.1 Screening data, eligibility and recruitment

The total number of included patients and reasons for non-completion will be summarized and tabulated. A trial profile flow diagram will be used to summarize the number of patients who were:

- Included at initial evaluation
- Eligible at initial evaluation and underwent installation of tympanostomy tubes.
- Received randomized allocation.

- Lost to follow-up.
- Randomized and included in primary analysis.
- Randomized and excluded from primary analysis.

7.2 Withdrawal/Follow-up

The status of eligible and randomized patients at trial end will be tabulated by treatment group according to whether they:

- Completed intervention but not assessments
- Withdrew consent.

7.3 Baseline patient characteristics

The patient demographics include age, gender, height, weight, tobacco use in the home, the educational, guardianship and work status of the parents/guardians. The patient demographics will be summarized and presented using descriptive statistics (N, mean, standard deviation, median) for continuous variables, and number and percentages of patients for categorical variables as appropriate.

8. Analysis

8.1 Analysis of the primary efficacy endpoint

8.1.1 Pure tone average (PTA)

The primary outcome will be the mean change in PTA from baseline between those patients controlled by their general practitioner and those controlled through the specialist health care system. PTA will be measured by taking the mean of the hearing thresholds in dB measured at 500Hz, 1kHz, 2kHz and 4kHz using pure tone audiometry.

8.1.2 Statistical analysis

Analyses will be performed according to intention to-treat. Mean +/- SD and summary statistics appropriate for the distribution will be reported for the primary outcome and each of the key secondary outcomes. A linear mixed model (LMM), with the combination of time and treatment as fixed factors (with three levels; 'baseline', 'post-ENT' and 'post-GP') and participant ID as random factor, will be applied to compare mean PTA 2 years after. We will also perform a t-test to compare the mean change in PTA after 2 years between GP controlled patients and those controlled within the specialist health care system.

8.1.3 Missing data

Missing data will not be imputed in the primary analysis as the statistical methods for handling missing data rely on untestable assumptions. If individual frequencies are lacking from the audiometric data, we will average the whole of the available registered frequencies to determine PTA. Baseline measurements of those lost to follow-up will be included for the linear mixed model analysis. Since there will be no data from the 2-year control, and therefore no measured difference, these will not affect the results of our t-test. Our goal is to minimize the amount of missing data to ensure the quality of our study.

8.2 Subgroup analyses

Subgroup analysis of the primary endpoint will be conducted for age, gender and severity of hearing loss at inclusion. The groups will be divided into thirds for analysis. Both the complete case set, and the two randomized groups will be divided into thirds according to level of hearing loss or age before subsequent analysis.

9. Safety analyses

Complications after implantation of ventilation tubes often occur outside of the standard controls. The current model refers the patient to their GP who can then refer the patient further to an ENT department should it be indicated. No changes to this practice were implemented.

10. Statistical software

All statistical analysis will be performed using Stata 18.0 MP (Stata corp. 2023. Stata statistical software: Release 18. College station, TX: Stata Corp LLC.) and R version 4.3.1 (R Core Team, R: A language and environment for statistical computing. R Foundation for Statistical Computing, Vienna, Austria. URL https://www.R-project.org/).

11. References

1. Austad B, Nilsen AH, Helvik AS, Albrektsen G, Nordgård S, Thorstensen WM. Postoperative controls of ventilation tubes in children by general practitioner or otolaryngologist? Study protocol for a multicenter randomized non-inferiority study (The ConVenTu study). *Trials* 2020; **21**(1): 950.
2. Browning GG, Rovers MM, Williamson I, Lous J, Burton MJ. Grommets (ventilation tubes) for hearing loss associated with otitis media with effusion in children. *Cochrane Database Syst Rev* 2010; (10): Cd001801.
3. Rosenfeld RM, Tunkel DE, Schwartz SR, et al. Clinical Practice Guideline: Tympanostomy Tubes in Children (Update). *Otolaryngol Head Neck Surg* 2022; **166**(1_suppl): S1-s55.
4. Vonen B. Dagkirurgi i Norge 2013-2017 [Ambulatory surgery in Norway 2013-2017]. *Ministry of Health and Care Services and Northern Norway Regional Health Authority* 2018; **3**: Report No.: 978-82-93141-34-1.
5. Isaacson GC. UpToDate: Overview of tympanostomy tube placement, postoperative care, and complications in children. 2023. https://sso.uptodate.com/contents/overview-of-tympanostomy-tube-placement-postoperative-care-and-complications-in-children (accessed Oct 2023).
6. Norsk forening for otorhinolaryngologi hode- og halskirurgi [Norwegian Society of Otorhinolaryngology Head and Neck Surgery]. Veileder for ørenese-halsfaget [Guideline for Otorhinolaryngology and Head & Neck Surgery]. 2011. http://legeforeningen.no/Fagmed/Norsk-Forening-for-Otorhinolaryngologi-Hode%2D%2DogHalskirurgi/Veileder-for-ore-nese-halsfaget/Otologi/Sekretorisk-otitt/.
7. Kay DJ, Nelson M, Rosenfeld RM. Meta-analysis of tympanostomy tube sequelae. *Otolaryngol Head Neck Surg* 2001; **124**(4): 374-80.
8. Derkay CS, Carron JD, Wiatrak BJ, Choi SS, Jones JE. Postsurgical follow-up of children with tympanostomy tubes: results of the American Academy of Otolaryngology-Head and Neck Surgery Pediatric Otolaryngology Committee National Survey. *Otolaryngol Head Neck Surg* 2000; **122**(3): 313-8.
9. Section for Applied Clinical Research. Randomization at NTNU. 2020. https://www.ntnu.edu/mh/akf/randomisering (accessed Oct 2023).

10. Austad B, Hetlevik I, Bugten V, Wennberg S, Olsen AH, Helvik AS. Implementing guidelines for follow-up after surgery with ventilation tube in the tympanic membrane in Norway: a retrospective study. *BMC Ear Nose Throat Disord* 2013; **13**: 2.
11. Austad B, Hetlevik I, Bugten V, Wennberg S, Olsen AH, Helvik AS. Can general practitioners do the follow-ups after surgery with ventilation tubes in the tympanic membrane? Two years audiological data. *BMC Ear Nose Throat Disord* 2014; **14**(1): 2.
