# Supplementary material for: Observation or Otolaryngology Surveillance After Ventilation Tube Insertion in Children: The ConVenTu Noninferiority Randomized Clinical Trial
Source: JAMA Otolaryngol Head Neck Surg. 2025 Oct 9;151(11):1063–70. doi: 10.1001/jamaoto.2025.2880 (PMC12512028; doi:10.1001/jamaoto.2025.2880)
Supplement: Supplement 2. — eAppendix. Information provided to general practitioners (GPs) and English translation eTable. Results of t testing of audiometric data and subgroup analysis [file jamaotolaryngolheadnecksurg-e252880-s002.pdf]

Supplemental Online Content

Yahiro R, Austad B, Helvik AS, Nilsen AH, Salvesen D, Thorstensen WM. Observation or otolaryngology surveillance after ventilation tube insertion in children: the ConVenTu noninferiority randomized clinical trial. *JAMA Otolaryngol Head Neck Surg.* Published online October 9, 2025. doi:10.1001/jamaoto.2025.2880

eAppendix. Information provided to general practitioners (GPs) and English translation

eTable. Results of *t* testing of audiometric data and subgroup analysis

This supplemental material has been provided by the authors to give readers additional information about their work.

© 2025 Yahiro R et al. *JAMA Otolaryngology-Head & Neck Surgery*.

eAppendix. Information provided to general practitioners (GPs) and English translation

ConVenTu-studien: Oppfølging hos fastlege
Denne pasienten deltar i en norsk studie om oppfølging etter innsetting av trommehinne-dren (ConVenTu-studien), og er randomisert til oppfølgende kontroller hos fastlege. Dette innebærer kontroll hos fastlegen 18 måneder etter operasjon samt tidligere ved behov. Pasienten/foresatte har fått beskjed om å bestille timer til dette selv.

På 18-månederskontrollen er det viktig å avgjøre om drenet sitter i trommehinnen eller ikke. Dersom det fortsatt sitter i trommehinnen, så henvis barnet til ØNH for kirurgisk fjerning.

Vedlagt er behandlingsforslag ved de vanligste problemene som kan oppstå etter innsetting av dren. Ved manglende behandlingsrespons eller andre problemstillinger kontakt behandlende ØNH-avdeling.

Redusert hørsel: Otoskopi (sitter drenet fortsatt i trommehinnen?), tympanometri hvis tilgjengelig: (1. Flat kurve og forhøyet øregangsvolum ved fungerende dren. 2. Flat kurve og normalt øregangsvolum hvis drenet har falt ut og det er residiv av væske i mellomøret, eller hvis drenet er tett,). Henvises til ny hørselstest og evt. ØNH-undersøkelse ved varighet >2 måneder.

Akutt mediaotitt: Medfører vanligvis rennende øre ved åpent dren i trommehinnen. Ved god allmenntilstand og sekresjon fra øret <3 dager kan tilstanden sees an uten behandling.

Rennende øre>3 dager: Bakterieprøve fra øregangen før behandlingsstart. Dersom mistanke om bakteriell infeksjon gis antibiotika øredråper. Førstevalg er Terra-Cortril Polymyxin B, 3 dråper x 3 i 7 dager. Behandling justeres etter resistensbestemmelse.

Dersom drenet har falt ut og barnet plages med residiverende otitter: henvis ny ØNH undersøkelse.

Vedrørende lokal antibiotikabehandling og ototoksisitet: Ved drensbehandling eller hull i trommehinnen vurderes lokalbehandling med Terra-Cortril Polymyxin B eller ciprofloxacin-preparater (Cilox, Cetraxal, Cetraxal comp) trygt. Ved øvrige lokale antibiotika og antimykotika anbefales konferering med ØNH-lege.

ConVenTu-Study: GP Follow-up (English Translation)
This patient is taking part in a Norwegian study regarding follow-up after the insertion of ear-drum ventilation tubes (the ConVenTu study), and is randomised to follow-up through their GP. This entails control 18-months after surgery through their GP and earlier upon request. The patient has been informed that they must book this appointment themselves.

The 18-month control is important to determine whether the ventilation tube is still in place. If the ventilation tube is still in place, refer the child to ENT for surgical removal.

Find attached treatment recommendations for the most common problems which can occur after ventilation tube insertion. Contact the responsible ENT department should the patient fail to respond to treatment or should other problems arise.

Hearing loss: Otoscopy (Is the ventilation tube still in place?), tympanometry if available: (1. Flat curve with high volume if the ventilation tube is functional. 2. Flat curve and normal volume if the ventilation tube has fallen out, and if there is a recurrence of fluid in the middle ear, or if the ventilation tube is obstructed. Referral to new audiometry and ENT examination if complaints persist >2 months.

Acute otitis media: Usually results in otorrhea if the ventilation tube is unobstructed. Watchful waiting if the patient is in good shape and secretion has lasted <3 days.

Otorrhoea >3 days: A test for bacteria from the ear canal should be taken before treatment. If bacterial infection is suspected treatment with antibiotic ear-drops should be initiated. First line treatment is Terra-Cortril Polymyxin B, 3

© 2025 Yahiro R et al. *JAMA Otolaryngology-Head & Neck Surgery*.

drops x3 for 7 days. Treatment should be adjusted according to the results of antibiotic resistance analysis when available.

If the ventilation tube has fallen out and the child is complaining of recurrent otitides: Re-referral to ENT examination.

Regarding local antibiotic treatment and ototoxicity: Terra-Cortril Polymyxin B and Ciprofloxacin (Cilox, Cetraxal, Cetraxal comp) are considered safe in the treatment of patients with ventilation tubes or perforation. Contact with ENT is recommended should other local antibiotics or antimycotics be required.

© 2025 Yahiro R et al. *JAMA Otolaryngology-Head & Neck Surgery*.

eTable. Results of *t* testing of audiometric data and subgroup analysis

**Audiometric Data (Per protocol group)**	**Observation (N=145)**	**ENT follow up (N=145)**	**Total**	**p value**
PTA at Inclusion (dB)	24.30 (8.26)	24.91 (8.87)	24.61 (8.56)	
PTA at Conclusion (dB)	12.19 (7.43)	12.47 (7.57)	12.33 (7.49)	0.75
Change in PTA	-12.11 (9.73)	-12.44 (10.48)	-12.28 (10.09)	0.78
**PTA at control (dB) by Age in years at Inclusion**	Mean (95%CI)	Mean (95%CI)	Difference in Means (95%CI)	
1st tertile 3.0-4.3 Years (N=99)	14.38 (12.38 to16.37)	14.14 (12.00 to 16.28)	0.23 (-2.66 to 3.13)	0.87
2nd tertile 4.4-5.8 Years (N=95)	11.89 (9.74 to 14.04)	11.89 (9.62 to 14.16)	0 (-3.09 to 3.09)	1.00
3rd tertile 5.8-11.8 Years (N=96)	10.41 (8.24 to 12.58)	11.39 (9.28 to 13.51)	-0.99 (-3.98 to 2.01)	0.52
**PTA at control by Sex**				
Males (N=174)	11.66 (10.14 to 13.18)	12.13 (10.65 to 13.60)	-0.47 (-2.58 to 1.65)	0.66
Females (N=116)	13.13 (11.04 to 15.21)	12.92 (10.76 to 15.08)	0.21 (-2.80 to 3.21)	0.89
**PTA at control (dB) by PTA at Inclusion**				
2.5 to 20 dB (N=103)	9.80 (8.18 to 11.42)	10.24 (8.77 to 11.71)	0.45 (-2.60 to 1.71)	0.68
20.6 to 28.8 dB (N=97)	12.35 (10.41 to 14.30)	13.39 (10.85 to 15.93)	-1.04 (-4.14 to 2.06)	0.51
29.2 to 46.9 dB (N=90)	14.85 (12.09 to 17.61)	14.13 (11.66 to 16.61)	0.71 (-2.93 to 4.36)	0.70

Data are n (%) or mean (SD) unless otherwise specified. PTA=Pure Tone Average. dB=decibels. GP=General Practitioner. ENT=Otorhinolaryngology.

© 2025 Yahiro R et al. *JAMA Otolaryngology-Head & Neck Surgery*.
