# Supplementary material for: Observation or Otolaryngology Surveillance After Ventilation Tube Insertion in Children: The ConVenTu Noninferiority Randomized Clinical Trial
Source: JAMA Otolaryngol Head Neck Surg. 2025 Oct 9;151(11):1063–70. doi: 10.1001/jamaoto.2025.2880 (PMC12512028; doi:10.1001/jamaoto.2025.2880)
Supplement: Supplement 3. — Data Sharing Statement [file jamaotolaryngolheadnecksurg-e252880-s003.pdf]

Data Sharing Statement

Yahiro. Observation or Otolaryngology Surveillance After Ventilation Tube Insertion in Children. *JAMA Otolaryngol Head Neck Surg*. Published October 09, 2025.
doi:10.1001/jamaoto.2025.2880

Data
Additional Information: ClinicalTrials.gov, www.clinicaltrials.gov, number NCT02831985.
Data available: No
